# Personality Traits and Urinary Symptoms Are Associated with Mental Health Distress in Patients with a Diagnosis of Prostate Cancer

**DOI:** 10.3390/curroncol28040262

**Published:** 2021-08-06

**Authors:** Charles Gillis, Gabriela Ilie, Ross Mason, Gregory Bailly, Joseph Lawen, David Bowes, Nikhilesh Patil, Derek Wilke, Robert David Harold Rutledge, David Bell, Ricardo Rendon

**Affiliations:** 1 Department of Urology, Dalhousie University, Halifax, NS B3H 4R2, Canada; ch412600@dal.ca (C.G.); Ross.Mason@Dal.Ca (R.M.); gbailly@dal.ca (G.B.); Joseph.Lawen@Dal.Ca (J.L.); David.Bell@Dal.Ca (D.B.); Ricardo.Rendon@Dal.Ca (R.R.); 2 Department of Community Health and Epidemiology, Dalhousie University, Halifax, NS B3H 4R2, Canada; 3 Department of Radiation Oncology, Dalhousie University, Halifax, NS B3H 4R2, Canada; David.Bowes@dal.ca (D.B.); Nikhilesh.Patil@nshealth.ca (N.P.); Derek.Wilke@nshealth.ca (D.W.); Rob.Rutledge@nshealth.ca (R.D.H.R.)

**Keywords:** mental health, prostate cancer, urinary problems, quality of life, survivorship, personality, neuroticism

## Abstract

Objective: With a prolonged natural history compared with many other cancers, prostate cancer patients have high rates of mental illness over the duration of their treatment. Here, we examine the relationship between personality and mental health distress in a sample of prostate cancer patients. Methods: This study was conducted in the Canadian Maritime provinces, where a cohort of 189 men with prostate cancer were invited to complete a quality-of-life online survey between May 2017 and December 2019. The presence or absence of screening positive for mental health illness was the primary outcome and was assessed using Kessler’s 10-item scale (K10). Urinary symptoms were assessed using the International Prostate Symptom Score (IPSS). The ten-item personality inventory (TIPI) assessed extraversion, agreeableness, conscientiousness, emotional stability (or neuroticism), and openness to experiences. A multivariate logistic regression model was created to examine the association between personality, urinary symptoms, and mental health distress, while controlling for time from diagnosis, treatment type, age, and multimorbidity. Results: Screening positive for mental illness (18.0%) was associated with personality traits of low levels of emotional stability (OR = 0.07, 95% CI: 0.03–0.20) and moderate to severe urinary problems (OR = 5.21, 95% CI: 1.94–14.05)). There was no identified association between treatment received for prostate cancer and personality type. Conclusion: Screening for mental health illness in this population may help reduce morbidity associated with cancer treatment, as well as identify patients who may be at risk of mental health distress and could benefit from individualized mental health support services. These findings suggest that multidisciplinary care is essential for the management of these patients.

## 1. Introduction

With excellent prognostic rates compared with many other cancers, favoring about a 90% survival at 10 years, men with prostate cancer experience a prolonged survivorship period [1]. There are a number of cultural and psychosocial stressors that can affect prostate cancer patients through this extended timeframe, predisposing this population towards the development of mental health disorders [2,3,4]. Indeed, approximately 16% of men who are prostate cancer survivors will experience clinically significant depression at some point during their treatment, compared with a global prevalence of depression of 4.7% [5,6]. While the management of mental health illness among prostate cancer patients is critical in a holistic oncologic treatment approach, this area remains largely understudied. Treatment-related adverse effects, cancer-related symptoms, and family support systems, as well as individual personality factors and coping abilities, can all contribute to the development of depression, negatively affecting overall cancer outcomes [2,6].

Personality is described in terms of the Big Five framework, where specific personality traits are clustered into broad abstract domains, including extraversion, agreeableness, conscientiousness, emotional stability, and openness [7]. Personality has previously been examined in oncology patients by Rochefort et al. [8], who found that neuroticism (or low emotional stability) was associated with worse physical health and maladaptive health behaviours. Prostate cancer patients with low optimism report high emotional distress [9]. Quality of life indicators have also been previously associated with personality. Positive aspects of well-being were found to be associated with personality traits while negative aspects of well-being were associated with personality traits and coping strategies among prostate cancer survivors, between one to eight years post-treatment [10]. Higher conscientiousness was associated with improved measures of physical health and better health behaviours. Neuroticism has been previously identified as being a particularly important personality trait to the response of an individual to health challenges, and may describe an individual’s limited resilience to negative stressors [11]. Neuroticism can cause increased feelings of irritability, worry, and decreased confidence in one’s ability to deal with the situation [12]. Patients high in neuroticism are theorized to have a heightened negative reaction to stimuli, leading to worse subjective symptom scores and greater stress when facing complications and bad news with regards to their health status [11,12,13]. Deimling et al. [14] found that neuroticism was a predictor of cancer-related worry, as well as the strongest predictor of depression in oncology patients.

Personality may play a strong role in identifying those patients at risk of experiencing significant psychologic distress, and services may be specifically offered for patients in the interest of paving the way towards multidisciplinary, individualized oncology survivorship care [15]. A recent study associated high neuroticism with the choice of surgery among prostate cancer patients [16]. However, the study had several methodological limitations, including that only certain questions of the personality scale were administered, thus potentially tampering with the personality scale’s reliability and validity. To our knowledge, this is the first study to assess the role of personality type in mental health outcomes among prostate cancer patients while considering the contribution of urinary symptoms, a known independent risk factor for mental distress in this population. To extend the literature, we also assessed the association between personality type and the type of treatment received for the prostate cancer diagnosis.

## 2. Methods

This study analyzed 189 men (mean age = 66.39 years), diagnosed with localized prostate cancer, who were surveyed between May 2017 and December 2019 as part of a Canadian Maritime survey examining the quality of life of prostate cancer survivors. Eligible patients had a diagnosis of localized prostate cancer, spoke English, resided in the Maritime provinces at the time of the survey, and had a valid email address. Patients were identified through urology and radiation oncology clinics and were sent a link for the survey in the mail. Among the patients at six clinic sites who were provided with a survey link, 68% responded. The survey length was approximately 25 min and could be completed online. Patients were able to access the study online through their own electronic devices, or a device was able to be provided to them in these settings. Study data were kept in an online database through the REDCap web application (Research Electronic Data capture) supported by the regional health authority. Patient consent was obtained electronically at the time of survey access. The study was approved by the Nova Scotia Health Authority Research Ethics Board.

### 2.1. Measures

#### 2.1.1. Mental Health Illness Assessment

Mental health illness was screened through the Kessler Psychologic Distress scale (K10), a well-validated, ten-item Likert-style survey that screens for distress symptoms such as depression and anxiety [17]. The K10 results in an overall score from 10 to 50, where 20–24 indicates mild mental health illness, 25–29 indicates moderate mental health illness, and above 30 indicates severe mental health illness. The K10 has been established as a valid tool for assessing clinical levels of mental health illness and has an 86% sensitivity and 83% specificity for a cut-off point of 20 [15]. Cronbach’s α for this scale in our sample was 88. Cronbach’s alpha for this scale reported elsewhere is also 88 and indicates a high level of consistency [18]. A categorical variable was created and used in all analyses, where 0 was coded to indicate a score below 20 and 1 was coded to indicate a score greater than 20 [18].

#### 2.1.2. Personality Traits Assessment

Personality was assessed through the Ten-Item Personality Index (TIPI) scale. The Big-Five framework model of personality identifies five domains of personality: extraversion, agreeableness, conscientiousness, emotional stability (or neuroticism), and openness to experiences [7]. The Big-Five model of personality can be approximated by small item inventories, such as the TIPI, where respondents assign a Likert score (1 = disagree strongly, 7 = agree strongly) to 10 statements relating to the five domains of personality. For the logistic regression analyses, each individual personality score was compared to the average in the general population and dichotomized (0 for scoring below the average in the population for the specific trait, and 1 for scoring above the average) for ease of clinical interpretation consistent with similar studies [19]. Patients were then described as being extraverted if they had an above average extraversion score, for example. In general, the TIPI has substantial psychometric properties for measuring the Big Five personality traits in older adults in terms of convergent validity. Results reveal substantial significant convergent correlations (extraversion, *r* = 87; agreeableness, *r* = 70; consciousness, *r* = 75; emotional stability/neuroticism, *r* = 81; openness to experience, *r =* 65) [7]. The TIPI also demonstrated adequate six-week test–retest reliability (*r* = 72). Cronbach’s α has been reported to lie between 0.40 and 0.68, a typical finding in short scales. Our sample’s α was 0.46.

#### 2.1.3. Urinary Symptoms

Urinary problems were assessed using the International Prostate Symptom Score (IPSS), a well-validated clinical tool that asks about the bother of seven urinary symptoms [20]. Responses for each of the questions ranged from 0—not at all; 1—less than 1 in 5 times; 2—less than half the time; 3—about half the time; 4—more than half the time; to 5—almost always. Patients are considered mild if scoring between 1 and 7, moderate if 8–19, and severe if 20–35. This variable was coded 0 for scores at or below 7 and 1 for scores at or above 8. I-PSS has good validity and reliability (Cronbach’s α of 80), and it is used commonly for assessing urinary function among PCa patients and survivors [21]. Cronbach’s α for the I-PSS in this sample was 86.

#### 2.1.4. Treatment Modality

Survey respondents were asked to indicate if they had (coded 1) or not (coded 0) received either radical prostatectomy, radiation (external beam or brachytherapy), or hormonal therapy (injections, pills, or an orchiectomy) for their prostate cancer diagnosis in the past, of if they were currently on active surveillance.

#### 2.1.5. Covariates

Age, education (coded 1 for completed high school or less, 2 for completed bachelor degree or less, and 3 for completed Masters, PhD, or MD), relationship status (coded 1 for married, living with a partner, or dating and coded 0 for widowed, divorced, separated, or never married), past year household income (coded 1 for <30 K, 2 for 30–69 K, 3 for 70 K to 100 K, 4 for >100 K), number of months of survivorship since diagnosis, current intake of any prescribed medication for depression (coded 0 for no and 1 for yes), comorbidities (none, coded 0, one, coded 1, two or more, coded 2), and employment status (unemployed or retired, coded 0, working part or full time, coded 1) were demographic variables describing the sample. Age, survivorship time and multimorbidity were covariates included in the final analysis.

### 2.2. Statistical Analyses

All analyses were performed with SPSS V25 (IBM Corp., 2017, Armonk, NY, USA). Frequency analyses described the characteristics of the sample. Logistic regression analyses assessed the relationship between personality type and each of the possible treatment types received for the prostate cancer diagnosis. To adjust for the possibility of an inflated experiment-wise error, Bonferroni’s adjustment was used to assess statistical significance (α = 0.01; 0.05/5 logistic regressions). A multivariate logistic regression analysis was used to model the association between the outcome, mental health distress and each of the seven predictors (severity of urinary symptoms, extraversion, introversion, agreeableness, conscientiousness, emotional stability/neuroticism, and openness to experience) and three covariates (age, survivorship time, multimorbidity). Prior to conducting the analyses, the assumptions of logistic regression were checked and found to be tenable. The primary outcome had 3.2% of missing data. After listwise deletion, the analytical sample for the multivariate logistic regression was 183.

## 3. Results

A total of 18% (*n* = 33) of men screened positive for mental health illness, and 3.7% reported currently being prescribed medication for depression, anxiety, or both. The mean age of respondents was 66.4 years (±6.8 years) with an average time between diagnosis and completion of survey of 23.4 months (±40.4 months). Most men in this sample have completed a bachelor degree or more (87.4%), they were retired or unemployed (65.9%), were currently in a relationship (either married, dating, or living with a partner) (92.9%), and their household income in the past year was over 70 K (46.2%). Most men in this sample had one or more additional comorbidities to their prostate cancer diagnosis (82.1%) and had mild urinary tract symptoms (60.3%). Men in this sample were above population averages on agreeableness (54.5%), conscientiousness (74.1%), and emotional stability (69.8%). Most men in this sample received active treatment(s) for their prostate cancer diagnosis. Table 1 describes the characteristics of the sample. Logistic regressions assessed the relationship between each treatment modality and personality trait. The results reveal no statistically significant relationships, *p* > 0.05 (see Table 2).

The fitted multivariate logistic regression assessing the presence or absence of positive screening results for mental health illness examining the contribution of the six predictors (severity of urinary symptoms, extraversion, introversion, agreeableness, conscientiousness, emotional stability/neuroticism, and openness to experience) while controlling for the three covariates (age, survivorship time, multimorbidity) was statistically significant *X*^2^(10) = 49.89, *p* < 0.001, with a Nagelkerke’s R^2^ = 0.39, and the Hosmer and Lemeshow test showing model stability (*X*^2^(8) = 5.84, *p* > 0.05). This model was accurate for nearly 86.2% of the individuals in this sample.; Table 3 reports Wald Chi-square statistics for each of the predictors in the model, their mean estimates and odds ratios. When all other variables in the model were held constant, moderate to severe urinary symptoms resulted in a statistically significant 5.21 (95% CI: 1.94–14.05) times higher odds for the presence of mental health illness. Screening positive for mental illness was associated with low levels of emotional stability (OR = 0.07 (95% CI: 0.03–0.20).

## 4. Discussion

Data on the contribution of personality traits to mental health illness in prostate cancer survivors are limited. Here, we investigated the relationship between personality type, urinary symptoms and mental health in a population of men with a history of prostate cancer, while controlling for age, survivorship time, and multimorbidity. Multivariate logistic regression modeling found that moderate to severe urinary symptoms and low emotional stability were statistically significantly associated with screening positive for current mental health illness.

The percentage of prostate cancer patients in this study screening positive for mental distress (18%) was similar to that reported in previous studies. A meta-analysis of 27 studies found that post-treatment prostate cancer patients had a prevalence of anxiety and depression of 18.4% [22]. This is in keeping with a large Canadian study indicating that 18.9% of prostate cancer patients screen positive for mild, moderate, or severe depressive symptoms [3,23,24]. Similarly, the finding of low emotional stability (i.e., neuroticism) as a predictor for mental health illness appears to be repeated in many oncology studies examining personality markers. A study by Perry et al. [19] examining prostate cancer survivors found significant associations between neuroticism and emotional distress (OR = 2.78), depression (OR = 4.23), and suicidal ideation (OR = 4.15). In their study, a high proportion of patients reported emotional distress (37%), which may be attributed to the use of an different mental health illness measure. Other studies reported lower rates of mental health illness, which are similar to our own results [2,3,15,25].

Neuroticism, being the inverse of emotional stability, is thought to describe the propensity of a person to experience negative emotions, especially when facing negative stimuli such as significant health stressors, and uncertainly around the diagnosis and what the treatment side effects may bring [26]. Neuroticism may play a role in the experience of adverse psychosocial and responses to physical side effects, such as social disconnect, isolation and anxiety around lack of erectile function [27]. Similarly, a study by Dahl et al. [16] provided evidence of increased adverse effects following robotic-assisted prostatectomy in patients with higher levels of neuroticism. This patient population was found to have high neuroticism levels (among 20% of the men in their sample), similar to our own finding (among 23.4% of the men in our sample). One limitation of the study by Dahl et al. is the exclusive use of only the neuroticism domain of the personality scale, rather than the employment of the entire measure as constructed, which might have tempered with the scale’s reliability and validity. Another limitation from Dahl et al study [16] is the lack of multiple treatment modalities groups. These findings suggest that patients with neuroticism either subjectively experience greater levels of emotional dysregulation following the advent of treatment-related side effects, or there is an inability to develop coping mechanisms to address this and potentially other persistent negative experiences or challenges following treatment. In our investigation, personality traits were not statistically significantly associated with any of the four treatment modalities (surgery, radiation, hormonal therapy, or active surveillance), consistent with other studies [28,29] which may suggest that this is cohort effect rather than one associated with a specific type of prostate cancer treatment. 

Although personality traits are considered to be conserved over time, oncology patients are subject to different stressors at multiple points during their survivorship journey, such as when experiencing new side effects, recurrences, or new treatment modalities to deal with possible recurrences. Another important consideration is that our patient population had a relatively short mean interval between diagnosis and survey (mean survivorship was 2 years, standard deviation was about 3 years) compared to the long survivorship journey most prostate cancer undertake. The true impact of mental illness in this population, and the associated contribution of personality, may not be truly captured in this study, as patients move on to require further treatments during their prostate cancer journey and may experience further treatment-related side effects. Regardless, as a result of the long survivorship time associated with prostate cancer, the successful management of mental health comorbidities may result in a significant gain in quality of life through years of treatment and should be considered to be incorporated in prostate cancer survivorship plans.

Other limitations to the current study are to be noted. This study is cross-sectional, and subject to significant recall bias and volunteer bias. Survival bias may limit generalizability, as those patients with significant comorbidities or severe disease may not be captured. Results indicate associations; thus, causality cannot be inferred. The sample size was relatively small. Since multimorbidity increases the risk of death from prostate cancer, the proportion of cases with high multimorbidity will be lower in our analysis, which could lead to an underestimation of the odds ratios. Future studies of larger sample sizes may consider evaluating the contribution of current disease status, the presence or absence of recurrence, time between treatments, recovery time post-surgery, and the interaction with other cancer diagnoses, which could play a significant role in the emergence of mental health illness in this population. Lastly, it is important to consider that we have no baseline pre-treatment data. It would be interesting and relevant to study how the personality factors and mental health screening evolve over time as patients progress through treatment and the survivorship period. It is a relevant negative that hormone therapy was not correlated with mental health outcomes, though that could change depending on whether the patient is actively on hormone therapy or if they were on it and their testosterone production has recovered.

Nonetheless, the results reported here provide an important contribution to our understanding of the association role played by personality to mental health illness, in a model where urinary function, multimorbidity, survivorship time, and age are assessed together. Screening for mental health illness and urinary symptoms is crucial in management of the prostate cancer patients, particularly those experiencing urinary morbidity, and there may be some additional benefit to identifying those patients with certain personality traits who may be worse off and may be experiencing mental distress and troublesome adverse psychosocial and physical effects [24]. This information is useful for clinicians and survivorship oncology teams because it points to the need to identify which patients or survivors may be at risk for experiencing mental health deterioration during the survivorship journey and points to the need for targeted therapies towards alleviating these potential stressors. An RCT performed by Shape et al. [30] demonstrated that, among oncology patients with depression, a multi-faceted collaborative approach can be more effective in alleviating mental distress symptoms for survivors. In another series, mindfulness-based approaches were found to improve stress levels and quality of life among breast cancer survivors. [31] Personalized health counseling or specific support groups may help in attenuating the negative response to psychosocial and physical stimuli associated with treatment side effects in neurotic patients. Further research is necessary to shed light on the causative relationship between personality and health outcomes, as well as developing interventions specifically targeted towards these patient groups [32].

## 5. Conclusions

There is a high prevalence of mental health disease in this cohort of patients in follow up after management of prostate cancer. Low emotional stability (neuroticism) may play a role in a maladaptive stress response to oncologic and oncologic survivorship care, predisposing these patients towards negative mental symptoms. Developing specific counseling services and other supportive measures targeted towards the management of stress for individual personality types may help reduce the morbidity of mental health concerns in prostate cancer patients.

## Figures and Tables

**Table 1:** Demographic characteristics of a sample of men with a history of prostate cancer diagnosis from the baseline cycle of the Soillse Prostate Cancer Quality of Life Maritimes Survey (2017–2019), *n* = 189.

Demographic Variable	Descriptive Statistics
*N* = 189	
Age (Mean, SD, *n*)	66.39 ± 6.79, 189
Number of months from diagnosis to completion of survey (Mean, SD)	23.35 ± 40.41, 189
Retired or unemployed, (%, *n*)	65.9 (182)
Currently in a relationship (%, *n*)	92.9 (182)
Income, % (n_i_/*n*)	
<30 K	7.1 (13,184)
30–69 K	46.7 (86/184)
70–99 K	39.1 (72/184)
>100 K	7.1 (13/184)
Education, % (n_i_/*n*)	
High school or less	12.6 (23/182)
Bachelor or less	40.1 (73/182)
Graduate education	47.3 (86/182)
Total health conditions, % (n_i_/*n*)	
None	17.8 (33/185)
One	60.5 (112/185)
Two or more	21.6 (40/185)
Screens positive for mental distress, K10, % (*n*/*N*)	18.0 (33/183)
Currently on medication for depression, anxiety, or both	3.7 (7/189)
Severity of lower urinary tract symptoms, IPSS, % (*n*/*N*)	
Mild	60.3 (111/184)
Moderate	35.9 (66/184)
Severe	3.8 (7/184)
Personality trait characteristic	
Extraversion, Mean ± SD, *n* % above population average, *n*/*N*	4.21 ± 1.59, 189 47.6 (90/189)
Agreeableness, Mean ± SD, *n* % above population average, *n*/*N*	5.28 ± 1.14, 189 54.5 (103/189)
Conscientiousness, Mean ± SD, *n* % above population average, *n*/*N*	5.93 ± 1.10, 189 74.1 (140/189)
Emotional stability, Mean ± SD, *n* % above population average, *n*/*N*	5.50 ± 1.25, 189 69.8 (132/189)
Openness to experiences, Mean ± SD, *n* % above population average, *n*/*N*	5.18 ± 1.20, 189 50.3 (95/189)
Treatment Modality	
Radical Prostatectomy, % (*n*/*N*)	51.9 (96/185)
External beam radiation or brachy, % (*n*/*N*)	45.9 (85/185)
Active surveillance, % (*n*/*N*)	9.2 (17/185)
Hormone therapy shots or pills, % (*n*/*N*)	48.6 (90/185)

**Table 2:** Logistic regression analyses assessing the role of personality traits in each treatment modality among men residing in Nova Scotia, aged 50–83, between 2017 and 2020, *n* = 185.

Presence or Absence of Each Treatment Modality	Radical Prostatectomy	Radiation Therapy	Hormone Therapy	Active Surveillance
	(*n* = 96, 51.9%) OR (95% CI)	(*n* = 84, 45.4%) OR (95% CI)	(*n* = 90, 48.6%) OR (95% CI)	(*n* = 17, 9.2%) OR (95% CI)
	*X*^2^(5) = 3.49	*X*^2^(5) = 1.31	*X*^2^(5) = 1.84	*X*^2^(5) = 8.12
Personality trait				
Extraversion	1.09 (0.83, 1.22)	1.07 (0.88, 1.29)	1.07 (0.89, 1.30)	0.75 (0.54, 1.05)
Agreeableness	0.84 (0.64, 1.12)	1.14 (0.87, 1.51)	1.15 (0.88, 1.52)	0.93 (0.58, 1.50)
Conscientiousness	1.06 (0.80, 1.40)	0.99 (0.75, 1.31)	0.96 (0.73, 1.28)	1.87 (0.96, 3.64)
Emotional stability	0.97 (0.75, 1.24)	1.00 (0.78, 1.29)	1.01 (0.79, 1.30)	1.06 (0.67, 1.66)
Openness	1.19 (0.91, 1.54)	0.96 (0.74, 1.24)	0.92 (0.70, 1.17)	0.86 (0.56, 1.34)

**Table 3:** Multivariate Logistic Regression analysis predicting mental health screening results by personality types, urinary symptoms severity while controlling for age, survivorship time, multimorbidity, among adult men residing in Nova Scotia, aged 50 to 83 years old, between 2017 and 2019, *n* = 183.

Variable in the Model	No Mental Disorder (*n* = 150) 1.0 Reference	Screening Positive for a Mental Disorder (*n* = 33) OR (95% CI)	Wald Chi-Square
			*X*^2^(9) = 48.64 ***
Age, Mean, SD	66.57 ± 6.80	65.79 ± 7.42 1.01 (0.94, 1.08)	*X*^2^(1) = 0.01
Survivorship time (months)	23.53 ± 42.64	18.06 ± 28.27 1.00 (0.99, 1.02)	*X*^2^(1) = 0.01
Multimorbidity			*X*^2^(2) = 1.47
None	18.7	9.1 1.0 Reference	
One	60.7	63.6 2.69 (0.38, 13.37)	*X*^2^(1) = 1.46
Two or more	20.6	27.3 2.23 (0.38, 13.13)	*X*^2^(1) = 0.79
Severity of lower urinary tract symptoms, IPSS, percentage			*X*^2^(1) = 10.66 **
Mild	65.4	36.3 1.00 Reference	
Moderate to Severe	34.6	63.7 5.21 (1.94, 14.05) **	
Extraversion	4.19 ± 1.53	4.24 ± 1.79 1.22 (0.46, 3.24)	*X*^2^(1) = 0.16
Agreeableness	5.42 ± 1.02	4.62 ± 1.39 0.59 (0.23, 1.53)	*X*^2^(1) = 1.17
Conscientiousness	5.96 ± 1.06	5.64 ± 1.29 0.81 (0.28, 2.37)	*X*^2^(1) = 0.14
Emotional stability	5.74 ± 1.13	4.33 ± 1.20 0.07 (0.03, 20) ***	*X*^2^(1) = 25.79 ***
Openness	5.21 ± 1.16	4.97 ± 1.35 1.08 (0.41, 2.86)	*X*^2^(1) = 0.02

** *p* < 0.01; *** *p* < 0.001.

## Data Availability

Data from this study are available to re-searchers through a data access process.
